# Artificial intelligence: a new editor limiting self-citation malpractice

**DOI:** 10.1186/s13054-023-04601-8

**Published:** 2023-08-29

**Authors:** Filippo Sanfilippo, A. Messina, F. Corradi, C. Robba

**Affiliations:** 1 Department of Anaesthesia and Intensive Care, A.O.U. Policlinico-San Marco, Catania, Italy; 2 Department of General Surgery and Medico-Surgical Specialties, School of Anaesthesia and Intensive Care, University of Catania, Catania, Italy; 3 Department of Biomedical Sciences, Humanitas University, Pieve Emanuele, Milan, Italy; 4 Department of Surgical, Medical, Molecular Pathology and Critical Care Medicine, University of Pisa, Pisa, Italy; 5 Department of Surgical Science and Diagnostic Integrated, University of Genoa, Genoa, Italy

Dear Editor in Chief—Prof. J.L. Vincent,

and

Dear Editor (self) Elect—Artificial Intelligence (AI),

We read with interest the letter reported by the two high-level researchers on behalf of the “self-electing” future Editor in Chief of the prestigious *Critical Care* journal [1]. As researchers, we agree that at some point we have to “*embrace the inevitable*” and that “*after all, resistance is futile*”. We also acknowledge that AI is not prone to the human imperfections and frailties. The scientific literature can certainly benefit from *“tireless, methodical, and impartial work”* produced by the AI, leading the medical community to the next Editorial step. However, this process is probably not as simple as the more enthusiastic supporters of AI may conceive; for instance, it has been widely reported that AI software may suggest wrong references [2], and same errors could happen during Editorial decision-making. Hence, the AI Editorial take-over may need a while, and the process will certainly need a gradual secondment with close supervision by people with great Editorial experience. The self-Elected Editor in Chief has to accept this graduality.

Nonetheless, we believe that in some aspects AI is already prepared to help the Editorial process, and such aspects should be embraced as opportunities by the Editors. For instance, an issue recently brought up is represented by the malpractice of author’s self-citation, which has been recently reported in anesthesiology and critical care medicine [3, 4], as well as in other fields [5]. Compulsory self-referencing represents a bad scientific practice resulting in artificial self-promoting. A self-reference has been defined as each time an article is cited by any of its co-authors. Landoni et al. found that self-citation attitude in anesthesiology and critical care medicine journals considerably increased from 11.5% (2006) to 21% (2007) and 44.4% (2008) [6]. Moreover, this phenomenon has further evolved in the creation of “citation farms”, where clusters of authors cite each other. Such approach is not harmless as it influences citation metrics making them spurious. These metrics are valued for exams, grants, and other competitions. Thereafter, this practice cannot be considered academically inoffensive, although it must be clear that some self-citations are fair and, in some cases, inevitable.

In order to restrict the malpractice of self-citations, it has been proposed to implement policies about self-citation, but not all journals have adopted such policies [3–5]. Moreover, it frequently happens that articles quotes references that are not truly supporting the statement in question. A recent study found that almost 40% of referencing errors were the citation of nonexistent findings, around 15% were incorrect interpretations of study findings, and 20% were chains of inaccurate citations copied from article to article [7].

It is certainly complex to tackle the phenomenon of self-citation. Certainly, asking the peer-review process to address also the issue of author’s self-referencing would be certainly too much, considering their work is done on voluntary basis and that priority is given to the critical evaluation of the manuscript quality over the appropriateness of references. Similarly, adopting a cut-off for author’s (and possibly journal’s) self-citations is unlikely to work considering the diversity of manuscripts (i.e. original, letters, reviews, etc.). An intriguing option could be to calculate authors’ scientific metrics excluding self-citations, and this approach would make inappropriate self-referencing useless. Importantly, Scopus^®^ and Web of Science^®^ databases offer such opportunity. However, if this approach is not pursued for any reason, the Editor (self) Elect AI may start its introduction in the future Editorial role as supervisor of appropriateness of references, building up an exceptional and fair barrier to inappropriate authors’ self-citation. Notably, the same process may be undertaken also for improving the practice of scientific journals, limiting the attention paid to the growth of impact factor (throughout journal’s self-citation) over the quality of the studies.

## Data Availability

Not applicable.

## Abbreviation

AI: Artificial intelligence
